# Evaluation of the Compressive Behavior of the Uniform and Graded Octet Lattice Cylindrical Shell Materials

**DOI:** 10.3390/ma19173605

**Published:** 2026-08-25

**Authors:** Hao Xu, Chengxuan Yu, Wenchang Luo, Weidong Cao, Xiaofei Cao, Chunwang He

**Affiliations:** 1 Key Laboratory of Theory and Application of Advanced Materials Mechanics, Department of Engineering Mechanics, School of Physics and Mechanics, Wuhan University of Technology, Wuhan 430070, China; 2 Research Center of Fluid Machinery Engineering and Technology, Jiangsu University, Zhenjiang 212013, China; 3 Institute of Advanced Structure Technology, Beijing Institute of Technology, Beijing 100081, China

**Keywords:** mechanical metamaterial, lattice cylindrical shell, mechanical properties, energy absorption, gradient design

## Abstract

Octet lattice cylindrical shell combines the stretching-dominated load transfer of Octet lattices with the geometric characteristics of the cylindrical shell, but the effects of different density gradients under different compression directions remain unclear. Uniform and three-layer graded 316L Octet LCSs were evaluated using quasi-static compression tests and validated finite element simulations. The results demonstrate that relative density is the primary factor controlling the overall stiffness, strength, and energy-absorption capacity of Octet LCSs. Under vertical compression, rearranging the density layers at a fixed average relative density regulates the yielding sequence and collapse path, enabling more controllable multistage energy absorption but with reduced stiffness and absolute SEA compared with uniform structures. Under transverse compression, the response is governed mainly by cross-sectional flattening, strut bending and local contact, and thus, the influence of layer arrangement on global load-bearing capacity is limited. The validated numerical model agrees well with the experiments and provides insights into the layer-sequence design of lightweight lattice cylindrical shells for protective and energy-absorbing applications.

## 1. Introduction

Lightweight structures are widely used in aerospace, transportation protection, marine engineering, biomedical implants, and energy-absorbing devices owing to their high specific stiffness, specific strength, and energy absorption characteristics. The classical work of Gibson and Ashby [1] established the fundamental relationships among the elastic response, yielding, and densification of cellular materials, while Maiti et al. [2] clarified their deformation and energy-absorption characteristics. Compared with stochastic foams, periodic lattices possess well-defined unit-cell topologies, controllable relative densities, and more predictable deformation paths. Deshpande et al. [3,4] demonstrated that stretching-dominated lattices primarily transfer loads through axial tension and compression, whereas bending-dominated lattices dissipate energy through strut bending, local buckling, and plastic deformation. These architectures can exhibit stable plateau responses and progressive collapse under quasi-static, impact, and blast loadings [5]. Advances in mechanical metamaterials and additive manufacturing have further enabled ultralight lattices with programmable properties and complex three-dimensional geometries [6,7,8]. Their mechanical performance is governed by unit-cell topology, relative density, and constituent material [9,10], while build orientation, strut dimensions, nodal geometry, surface quality, defects, and strain rate can also affect stiffness, strength, deformation, and energy absorption [11,12,13]. Graded lattices therefore provide an effective means of tailoring local stiffness, yielding sequence, load plateau, and energy-absorption efficiency by varying relative density, strut diameter, or unit-cell size [14,15,16].

Among various lattice topologies, the Octet lattice is a representative stretching-dominated space-frame architecture whose struts ideally carry axial tension and compression. Feng et al. [17] showed that its elastic isotropy and anisotropy can be regulated through geometric design. Zhang et al. [18] demonstrated the potential of grid Octet-truss configurations for energy absorption. Chen et al. [19] further found that the macroscopic yielding response of Octet lattices under multiaxial loading depends on relative density, loading path, strut orientation, and unit-cell connectivity. Although Octet lattices generally exhibit high specific strength and energy absorption under quasi-static and dynamic compression [20,21], their actual response may deviate from ideal axial load transfer because of nodal stress concentrations, local strut buckling, connection failure, and fillet geometry [22,23]. Comparisons among different lattice topologies also confirm that unit-cell architecture is a primary factor governing stiffness, strength, and energy-absorption efficiency [24]. For additively manufactured metallic lattices, dimensional deviations, surface roughness, and initial geometric imperfections can modify local stress distributions, yielding behavior, and failure paths [25,26,27]. Zhong et al. [28] demonstrated that 316L stainless steel is suitable for selective laser melting (SLM) because of its good processability, corrosion resistance, and ductility. Nevertheless, its microstructure, mechanical properties, and as-built relative density remain sensitive to manufacturing parameters and structural geometry [29,30]. Therefore, reliable investigation of 316L Octet lattices requires material characterization, repeated compression tests, and experimentally validated numerical models rather than idealized geometries alone [31].

Lattice cylindrical shells (LCSs) combine the geometric load-bearing characteristics of curved shells with the lightweight and tailorable properties of lattice structures. Under vertical compression, transverse compression or bending loadings, they may undergo coupled deformation involving strut buckling, cross-sectional ovalization, local contact, and densification. Wang et al. [32] investigated the compressive behavior of Gyroid LCSs and showed that curvature, unit-cell arrangement, and relative density jointly affect their mechanical response. Laskowska et al. [33] similarly demonstrated the energy-absorption potential of SLM-fabricated Diamond TPMS cylindrical structures. For graded BCC LCSs, Guan et al. [34] found that the density-layer distribution can change the yielding sequence and plateau response under vertical compression, while LCSs under bending exhibit cross-sectional deformation, local buckling, and plastic bending of the struts [35]. Comparisons among unit-cell topologies further indicate that topology, shell dimensions, and spatial mapping methods are important design variables [36]. Zhao et al. [37] showed that introducing density gradients and hybrid configurations can regulate crushing initiation and progressive collapse. Functionally graded, layered, and stiffness-tailored lattices can also improve strength, load-plateau stability, and energy absorption by controlling the local deformation sequence [38,39,40]. Dual-gradient, hierarchical, and bioinspired architectures further expand the design space for fatigue resistance, strength–toughness synergy, and multiobjective optimization [41,42,43], while hierarchical configurations, tapered struts, and auxetic lattices can enhance energy absorption by modifying local stiffness and failure modes [44,45,46].

Despite recent progress in lattice cylindrical shells, the effects of relative density, axial layer sequence, and loading direction on the mechanical response of stretching-dominated Octet LCSs remain unclear. To address this gap, uniform Octet LCSs with relative densities ranging from 0.09 to 0.25 and three graded Octet LCSs with the same average relative density of 0.17 were designed using the established cylindrical mapping framework of Wang et al. [32]. Unlike the LCS topology investigated by Wang et al. [32] and the graded BCC LCSs studied by Guan et al. [34], the present work focuses on the stretching-dominated Octet topology and its layer-sequence-dependent response under different compression directions. The three graded structures contained the same density set of 0.09, 0.17, and 0.25 and differed only in axial layer sequence, thereby isolating the effect of layer arrangement. Quasi-static compression experiments and validated finite element simulations were then used to reveal how the deformation mechanism changes from axial strut-dominated progressive collapse under vertical loading to cross-sectional flattening, strut bending, and local contact under transverse loading. The specific contribution of this study therefore lies in establishing the topology-specific effects of layer sequence and loading direction in graded Octet LCSs.

## 2. Design of Structure

**Figure 1:**
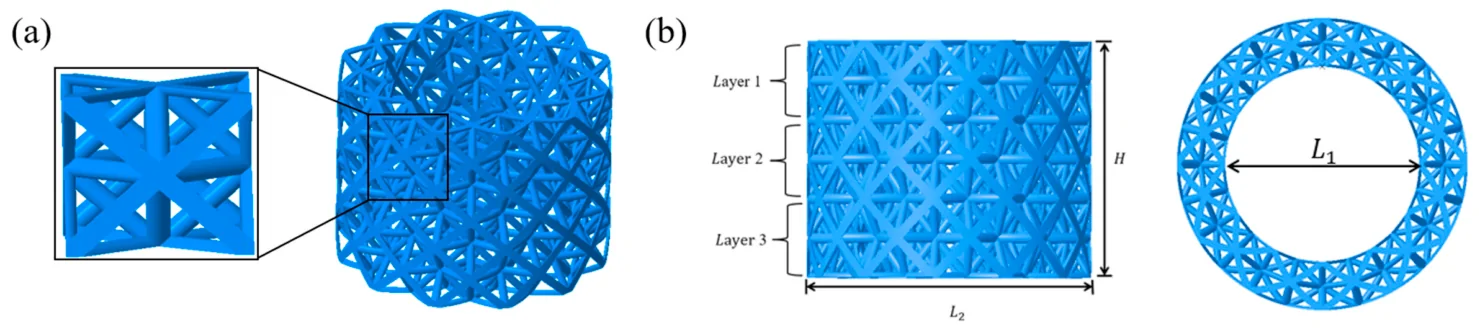
(**a**) Octet lattice cylindrical shell and representative unit cell. (**b**) Geometric parameters of the lattice cylindrical shell.

In this study, the Octet topology was adopted as the lattice unit cell. The Octet is a highly symmetric three-dimensional frame unit cell in which the struts primarily carry axial compression and tension rather than bending, thereby providing high stiffness and load-bearing capacity while maintaining good stability and relatively uniform deformation under multiaxial loading. Experimental and numerical studies of 316L Octet-truss and 316L metallic lattices have shown that unit-cell geometry, strut dimensions, manufacturing accuracy, and unit-cell orientation significantly affect lattice stiffness, strength, and deformation stability [47,48]. Following the cylindrical lattice-mapping method developed by Wang et al. [32], the Octet unit cell was mapped onto the cylindrical surface to construct the Octet LCS geometry. In the design, 12 lattice cells were arranged circumferentially and three cell layers axially. The cells were uniformly distributed within each layer to ensure a consistent mechanical response under compression. To obtain the desired mechanical properties, the strut diameter was varied among the layers to form graded lattice structures. Specifically, the uniform specimens had relative densities of 0.09, 0.13, 0.17, 0.21, and 0.25, whereas the graded specimens had an average relative density of 0.17. The key geometric parameters were the strut diameter D, inner diameter $L_{1}$, outer diameter $L_{2}$, and height H, all of which strongly influence the mechanical response of the specimens. The Octet unit cell, the cylindrical-shell mapping scheme, and the definitions of the geometric parameters are shown in Figure 1. Detailed dimensions of all structures, including the strut diameter in each layer, are listed in Table 1. All three graded designs used the same three axial-layer relative densities of 0.09, 0.17, and 0.25 and had the same average relative density of 0.17. Only the stacking sequence of these layers was varied. Therefore, the graded configurations were designed specifically to isolate the effect of layer sequence while keeping the density set, average relative density, and number of layers constant.

## 3. Experimental Test

### 3.1. Specimen Preparation

**Figure 2:**
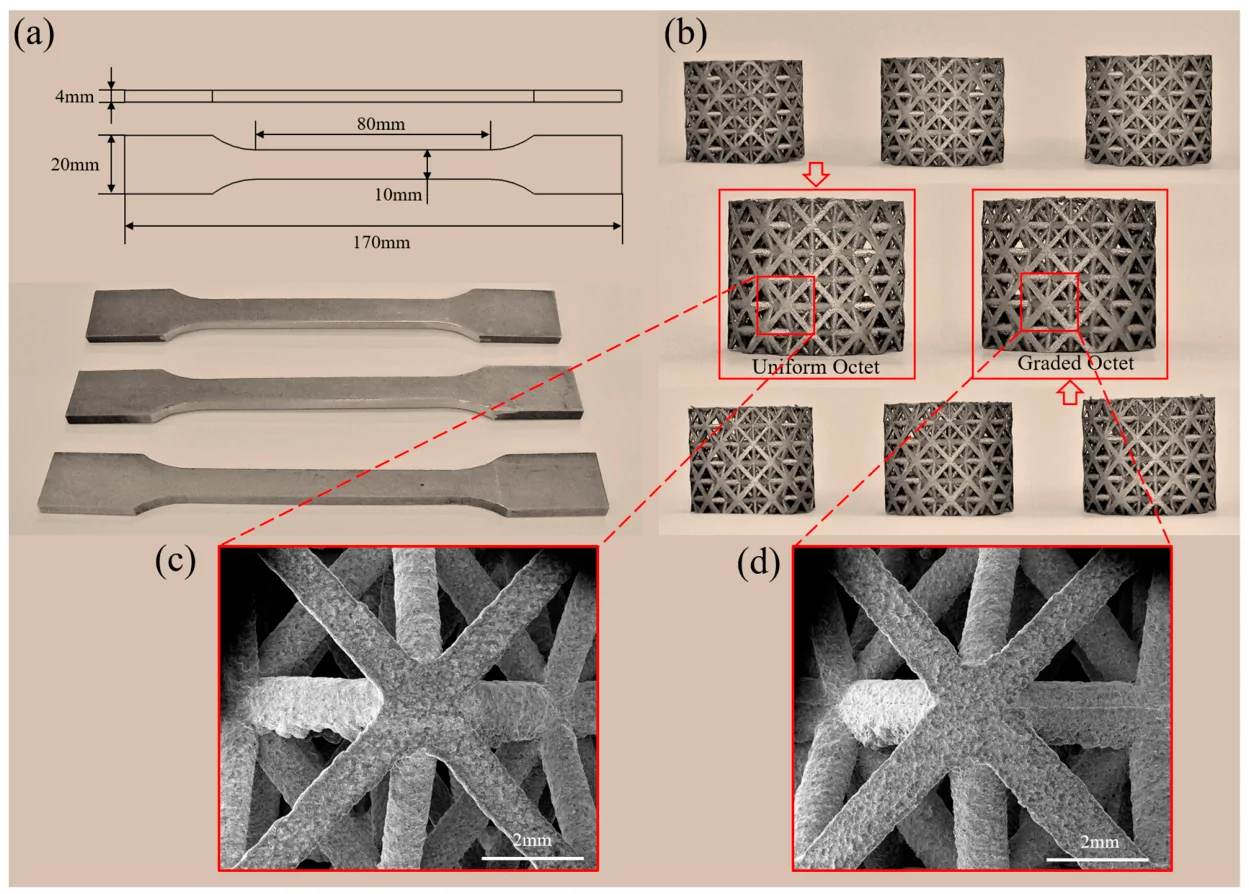
(**a**) Dimensions of the dog-bone shaped specimens for tensile testing. (**b**) Uniform and graded Octet LCS specimens. (**c**) Representative SEM image of the local strut and nodal morphology of the uniform Octet LCS. (**d**) Representative SEM image of the local strut and nodal morphology of the graded Octet LCS.

The LCS specimens were divided into uniform and graded groups. The uniform specimens had relative densities of 0.09, 0.13, 0.17, 0.21, and 0.25, whereas the graded specimens had an average relative density of 0.17, with different strut diameters in the individual layers to create the gradient and achieve the desired mechanical properties. The specimens were fabricated from 316L stainless steel powder by SLM using a Lim-X260A 3D printer (Tianjin LiM Laser Technology Co., Ltd., Tianjin, China). The laser power, scanning speed, and layer thickness were set to 350 W, 950 mm/s, and 0.06 mm, respectively, to ensure dimensional accuracy of the lattice geometry and struts. After printing, the specimens were surface-polished and annealed in an air furnace to reduce residual stresses and improve the as-built quality. Uniaxial tensile tests were also performed on dog-bone specimens to characterize the mechanical properties of the 316L stainless steel. During the tensile tests, the axial strain within the gauge length was measured directly using an extensometer attached to the gauge section in accordance with GB/T 2975-2018 [49], and the measured data were used to obtain the stress–strain response for material characterization. The designed dimensions and printed dog-bone specimens are shown in Figure 2a, and the uniform and graded lattice specimens are shown in Figure 2b. Three specimens of each type were fabricated to ensure experimental repeatability and satisfactory manufacturing quality.

The specimens were weighed after post-processing to determine their mass-based as-built relative densities. For the uniform and graded LCSs with a nominal relative density of 0.17, the design mass was 13.318 g, whereas the measured average masses for the as-built specimens were 12.623 and 12.652 g, respectively. The corresponding as-built relative densities were 0.1611 and 0.1615, representing reductions of 5.22% and 5.00% from the design value. These deviations are mainly attributed to variations in the as-built strut dimensions introduced during SLM and to material removal during surface post-processing. However, the measured masses of the uniform and graded specimens differed by only approximately 0.23%, indicating closely comparable actual material contents. Representative SEM images of the uniform and graded specimens are presented in Figure 2c and d, respectively, illustrating the as-built strut and nodal morphology. Considering these manufacturing-related variations, previous studies have shown that the unit-cell features, strut dimensions, actual relative density, surface roughness, and manufacturing defects of SLM-fabricated 316L lattices affect their compressive performance. Accordingly, repeated specimens, post-processing, and material tensile tests were used in this study to improve the reliability of the experimental data [48,50].

### 3.2. Quasi-Static Compression Test

Quasi-static compression tests of the LCS specimens were conducted on a 300 kN MTS E45.305 universal testing machine (MTS Systems, Eden Prairie, MN, USA). Quasi-static vertical compression tests were conducted at a crosshead speed of 1.5 mm/min, corresponding to a nominal axial strain rate of 0.001 s^−1^. The axial strain during tensile testing was measured directly using an extensometer attached to the gauge section to obtain the force–displacement responses. During testing, the deformation process was recorded at 30 frames per second using a SONY FDR-AX45 camera (Sony Corporation, Tokyo, Japan) to observe the deformation modes and failure characteristics. Before testing, all specimens were visually inspected to ensure intact surfaces and the absence of obvious defects.

**Figure 3:**
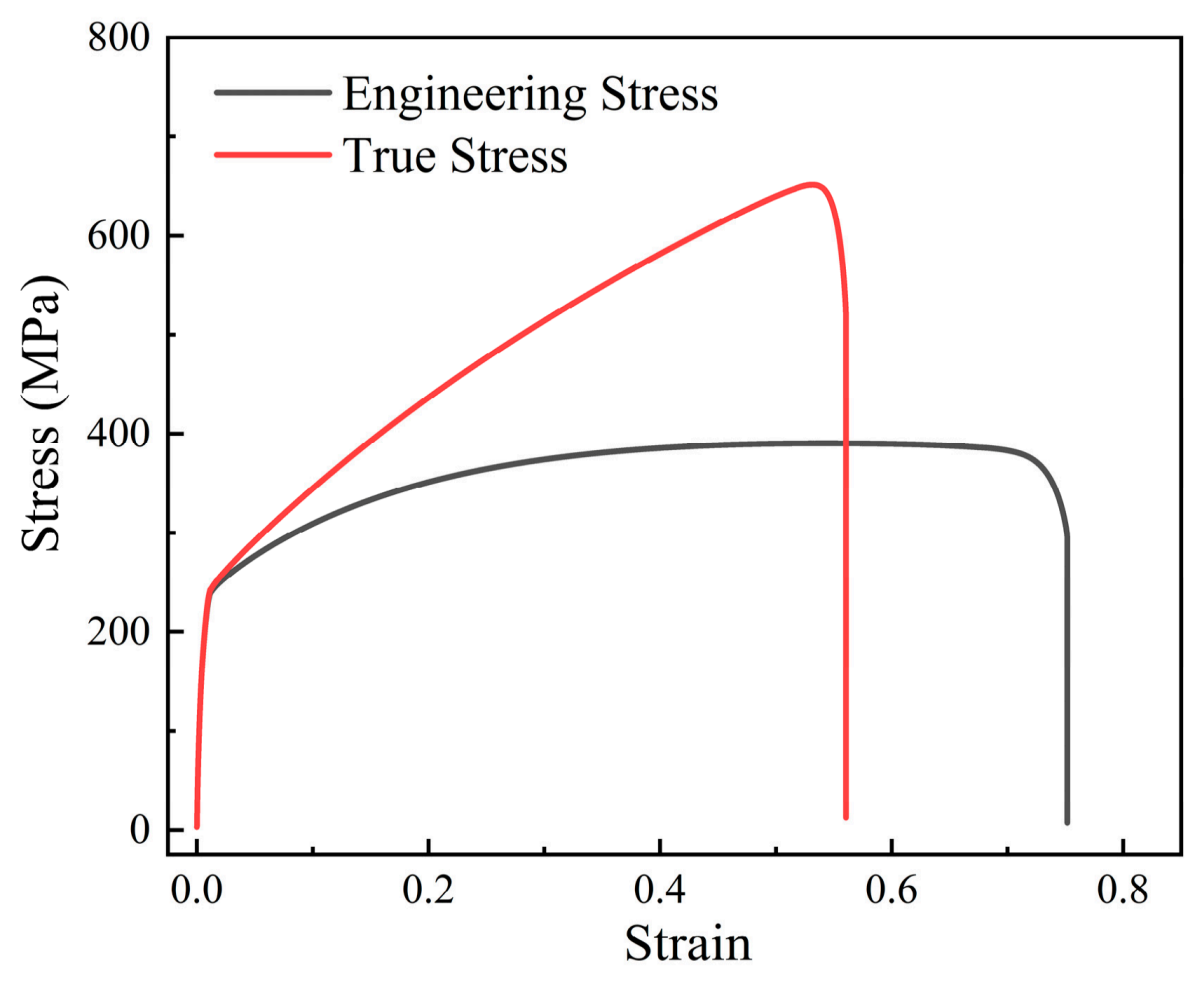
Average engineering and true stress–strain curves of the dog-bone specimens.

To characterize the mechanical properties of the 316L stainless steel, uniaxial tensile tests were performed on dog-bone specimens. Three repeated tests were conducted, and the corresponding engineering and true stress–strain data were averaged to obtain the mean curves shown in Figure 3. The three tests exhibited similar trends, indicating good repeatability and a stable material response. The averaged tensile response can be divided into three stages. In the initial elastic stage, the material responded linearly, with a Young’s modulus of 30.2 ± 1.2 GPa. Yielding and plastic deformation began at 242.6 ± 12.2 MPa. During strain hardening, the true stress continued to increase to an ultimate strength of 650.4 ± 39.8 MPa at an ultimate strain of 0.53, indicating substantial plastic deformation capacity before fracture. Overall, the mean curves provide a representative description of the tensile behavior of the 316L stainless steel. The relatively low Young’s modulus measured in this study may be related to the specific condition resulting from the SLM fabrication process, since differences in manufacturing equipment and processing conditions have been reported to cause considerable variations in the elastic response of additively manufactured 316L [51,52].

### 3.3. Experimental Results

**Figure 4:**
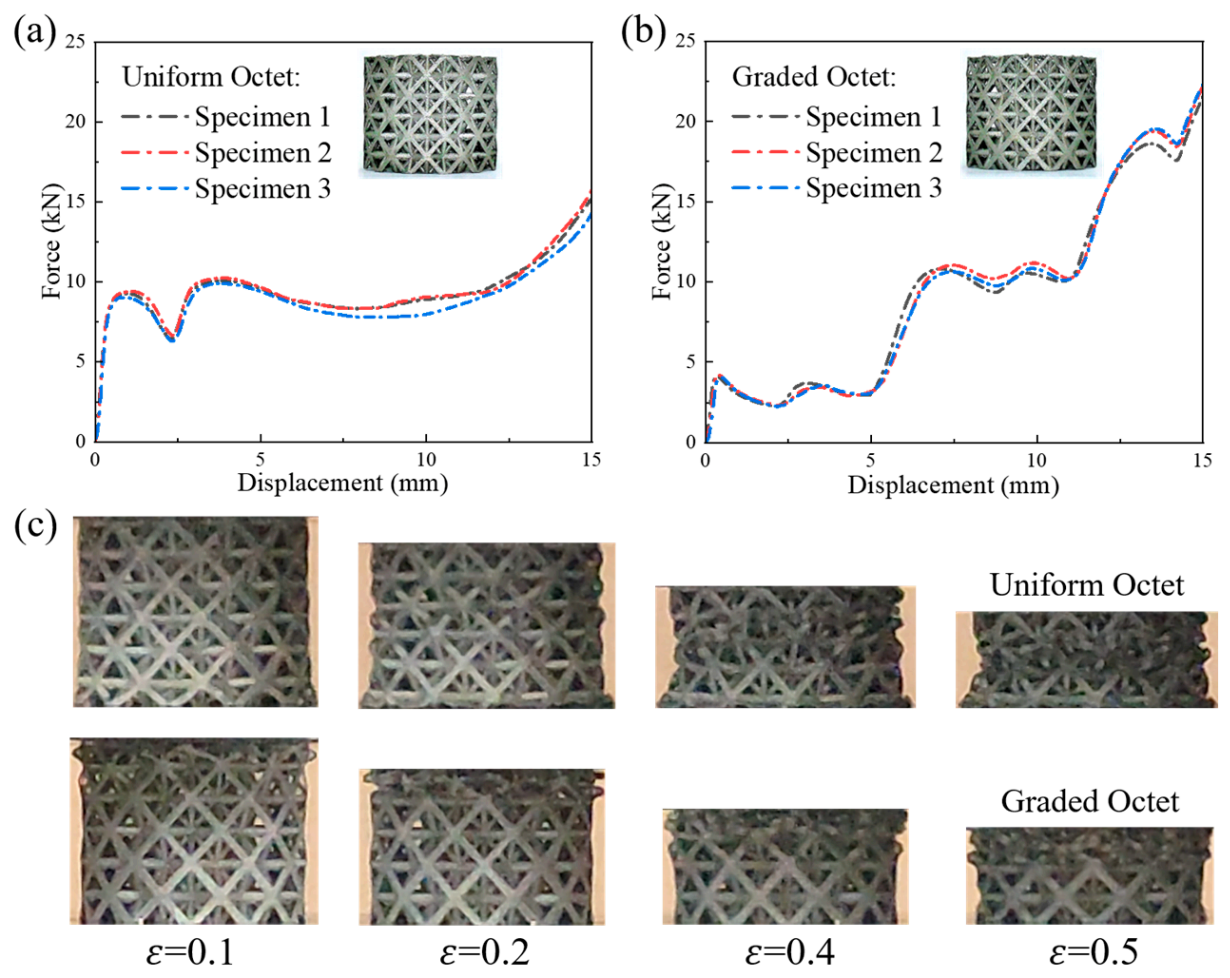
Experimental quasi-static compression force–displacement curves of (**a**) uniform Octet LCS specimens and (**b**) graded Octet LCS specimens. (**c**) Deformation processes of the Octet LCS specimens.

Figure 4a,b present the force–displacement curves and representative deformation processes of the uniform and graded Octet LCS specimens under quasi-static compression. For both specimen types, the curves from the three repeated tests nearly overlap and show similar trends during the initial load rise, plateau deformation, and subsequent rapid load increase, indicating stable manufacturing quality and good repeatability and reliability of the experimental data. For the uniform Octet LCS specimens, the load rises rapidly at the beginning of compression and enters a relatively stable plateau after yielding. During this stage, the lattice struts progressively undergo plastic deformation, local buckling, and collapse, allowing the structure to maintain a relatively stable load-bearing capacity over a large displacement range. With further compression, the lattice pores are progressively compacted and the struts come into contact and stack, causing the specimen to enter the densification stage. The overall stiffness then increases markedly and the load rises rapidly.

In contrast, the graded Octet LCS specimens exhibit a more pronounced staged compression response. Because the layers have different relative densities, their strut diameters and load-bearing capacities differ, and yielding therefore occurs sequentially rather than simultaneously, followed by layer-by-layer collapse. The low-density layer first yields and crushes at a relatively low load, the medium-density layer subsequently yields at a higher load, and the high-density layer, with its larger strut diameter and greater compressive resistance, yields and collapses last at the highest load. Consequently, three distinct load plateaus appear in the force–displacement curve of the graded lattice, corresponding to the progressive failure of the three density layers. The low-density layer has the lowest yield stress, followed by the medium-density layer, while the high-density layer has the highest yield stress. This low-to-high yielding sequence produces a smoother and more controllable deformation process. The deformation images at different compressive strains in Figure 4c also show that the uniform structure undergoes relatively synchronous global compression, whereas the graded structure exhibits clear layerwise collapse. This confirms that gradient design can effectively regulate the compressive deformation mode and energy-absorption behavior of lattice cylindrical shells. The layerwise failure mechanism is consistent with the sequential-collapse behavior commonly observed in functionally graded lattices [53].

## 4. Finite Element Analysis

### 4.1. Finite Element Model

**Figure 5:**
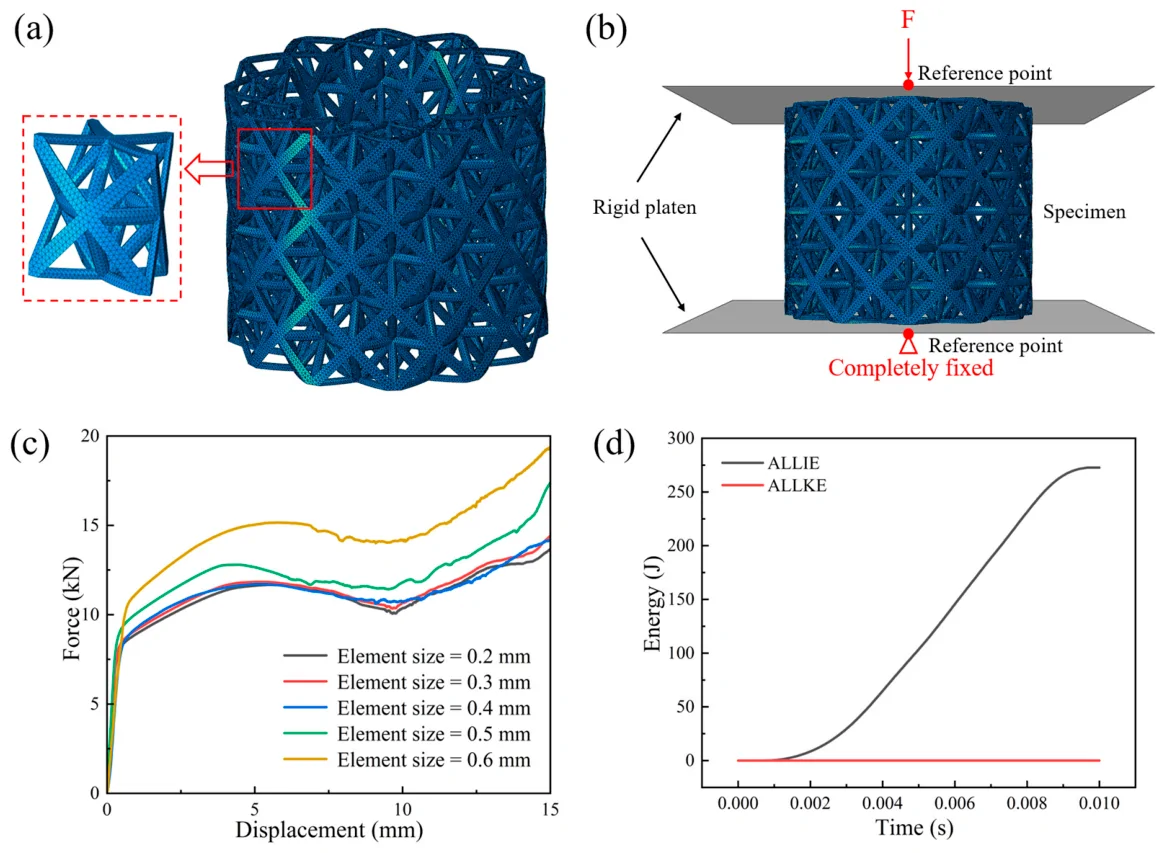
(**a**) Finite element model of Octet LCS. (**b**) Boundary conditions settings. (**c**) Mesh sensitivity analysis under different element sizes. (**d**) Energy balance during quasi-static compression.

To further investigate the mechanical response of Octet LCSs under quasi-static compression, a finite element model with the same geometry as the experimental specimens was established, as shown in Figure 5a,b. Finite element preprocessing was performed in HyperMesh (v2023), and a uniform mesh size was used throughout the lattice to ensure consistent discretization and comparable numerical results in different regions. The lattice struts were meshed with C3D10M ten-node modified quadratic tetrahedral elements, which are well suited to the curved surfaces and nodal connections of complex lattice geometries. A mesh-sensitivity analysis was conducted using five nominal element sizes of 0.2, 0.3, 0.4, 0.5, and 0.6 mm. As shown in Figure 5c, the force–displacement responses obtained using 0.4, 0.3 and 0.2 mm meshes were almost identical, while coarser meshes produced noticeable deviations. Therefore, a uniform element size of 0.3 mm was selected for subsequent simulations to balance computational accuracy and efficiency.

The elastoplastic material parameters obtained from tensile tests of the 316L stainless steel were used to describe plastic deformation during compression. The analyses were conducted in ABAQUS/Explicit v14 without mass scaling. The LCS specimen was placed between two rigid platens; the lower platen was fully fixed, and an axial displacement was applied to the upper platen through a reference point to reproduce the experimental compression process. Surface-to-surface contact was defined between the specimen and the platens, with hard contact in the normal direction and a penalty-friction formulation in the tangential direction. The coefficient of friction was set to 0.1. The energy histories were monitored to verify the quasi-static condition. As shown in Figure 5d, the kinetic energy (ALLKE) remained negligible compared with the internal energy (ALLIE) during most of the compression process, indicating that inertial effects had a negligible influence on the simulated response.

### 4.2. Comparison with the Experimental Results

**Figure 6:**
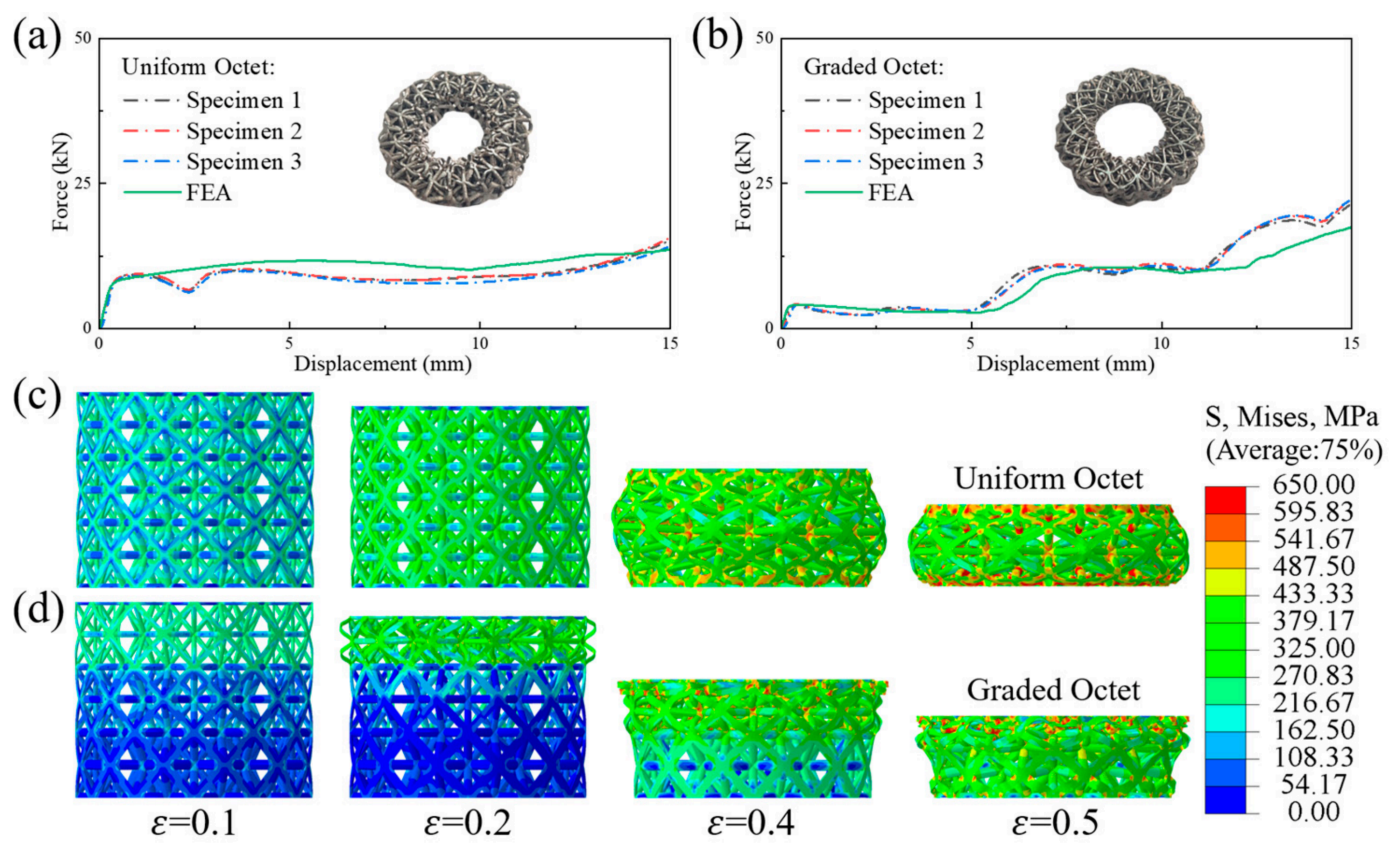
Comparison of the force–displacement curves of (**a**) uniform and (**b**) graded Octet LCSs between experiment and simulation. Simulated deformation processes and stress distributions of (**c**) uniform and (**d**) graded Octet LCSs.

Figure 6 compares the finite element and compression-test results, including the force–displacement curves, deformation processes, and stress distributions of the uniform and graded Octet LCSs at different compression stages. The finite element model predicts the overall quasi-static compressive response of both structures well. The simulated curves show trends similar to those of the three experimental curves during the initial loading, plateau deformation, and late densification stages. For the uniform Octet LCSs, the simulation reproduces the stable plateau after initial yielding. The specimen collapses relatively uniformly during compression and progressively enters densification as the displacement increases, accompanied by a rise in load. For the graded Octet LCSs, the simulation also captures the staged load-bearing behavior caused by sequential yielding of the density layers. The multiple plateau regions in the simulated curve are consistent with the experimental results, indicating that the model effectively describes the layer-by-layer collapse mechanism of the graded structure. The stress contours show that, at the beginning of compression, stresses are concentrated mainly at strut intersections and locally compressed struts. As the compressive strain increases, the high-stress regions expand, the struts deform plastically and come into contact, and a densified configuration ultimately develops.

To quantitatively evaluate the accuracy of the FE model, the simulated Young’s modulus, yield strength, and SEA of the uniform Octet LCS were 1714.5 MPa, 19.2 MPa, and 12.99 J/g, respectively, compared with the corresponding experimental values of 1781.3 ± 112.1 MPa, 20.5 ± 1.0 MPa, and 11.16 ± 0.94 J/g. The resulting relative errors were 3.75%, 6.35%, and 16.40%, respectively. For Graded design 1, the simulated values were 1176.1 MPa, 10.1 MPa, and 9.12 J/g, while the corresponding experimental values were 1127.6 ± 50.7 MPa, 10.3 ± 0.5 MPa, and 10.48 ± 0.72 J/g, giving relative errors of 4.30%, 2.24%, and 12.98%, respectively. Except for SEA, the relative errors of Young’s modulus and yield strength were all below 6.4%. The SEA errors were approximately 15% (16.40% for the uniform structure and 12.98% for Graded design 1). The comparatively larger SEA error is reasonable because SEA represents the cumulative response over the entire compression process and is more sensitive to SLM-induced dimensional deviations, surface roughness, local defects, the timing of layer collapse, contact, and densification [48,50,54]. These results indicate that the model accurately captures the initial stiffness and yielding response while providing a reasonable prediction of the overall energy absorption.

## 5. Results and Discussion

### 5.1. Vertical Loading Mechanical Properties

#### 5.1.1. Effect of Relative Density

**Figure 7:**
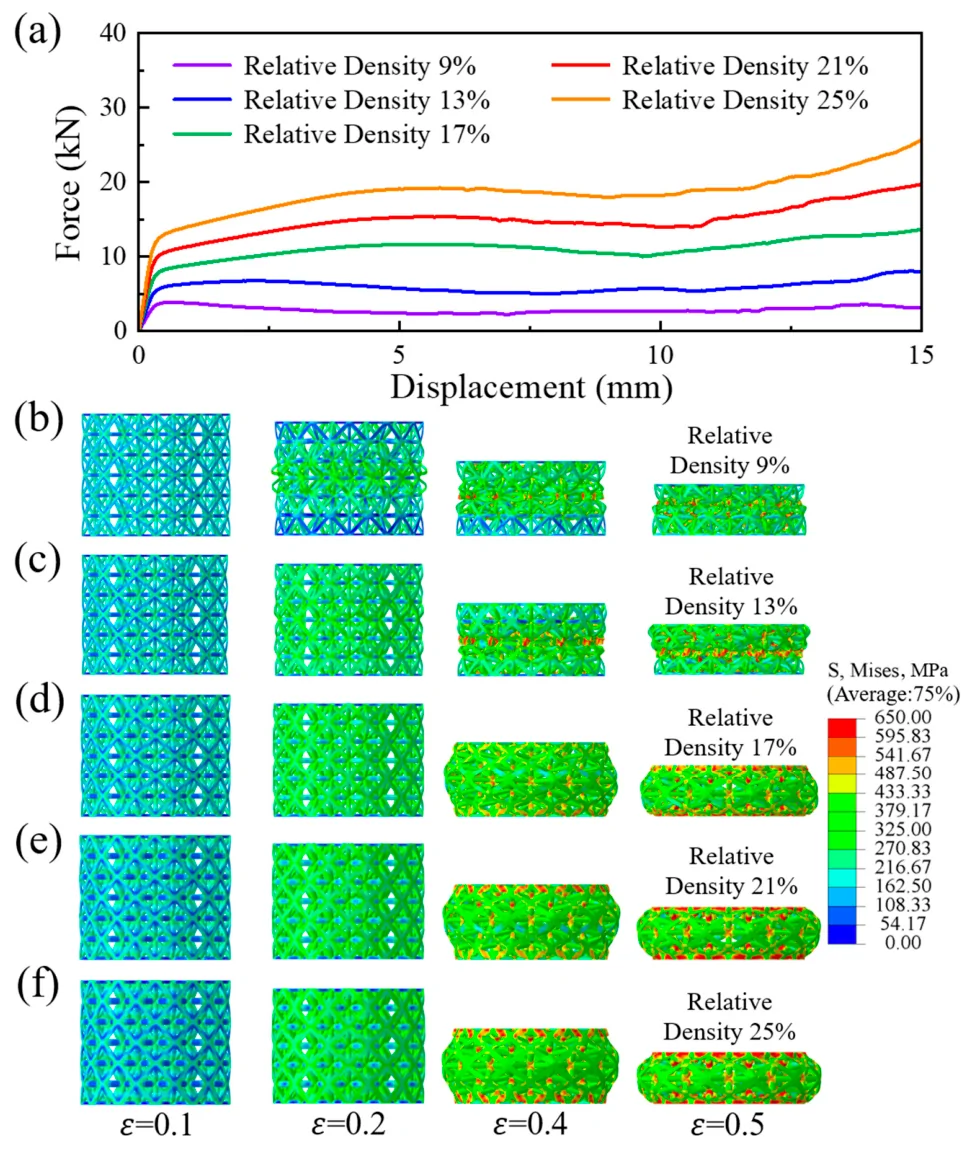
(**a**) Force–displacement curves and (**b**–**f**) deformation processes and stress distributions of the uniform Octet LCSs with different relative densities under vertical compression.

Figure 7 presents the force–displacement curves, deformation processes and stress distributions of the uniform Octet LCSs with different relative densities under vertical compression. As shown in Figure 7a, relative density has a pronounced effect on the vertical load-bearing capacity. As the relative density increases from 9% to 25%, the initial stiffness, yield load, and plateau load all increase markedly. This is primarily because a higher relative density corresponds to a larger strut diameter, which increases the volume fraction of load-bearing material and improves the resistance of the struts to bending and buckling, allowing the overall structure to withstand higher compressive loads. All specimens show a rapid load rise at the beginning of compression, followed by a relatively stable plateau, indicating that Octet LCSs can sustain load and absorb energy continuously during vertical compression. With further displacement, the curves rise gradually as the structures enter densification; the lattice pores close, the struts contact and accumulate, and the overall stiffness increases substantially.

The deformation processes and stress contours in Figure 7b–f show that specimens with different relative densities follow similar overall deformation modes. As the compressive strain increases, the structures gradually transition from elastic deformation to plastic buckling and global crushing and finally enter the densification stage. However, higher relative densities produce more extensive high-stress regions and higher overall stress levels, indicating that denser lattices can sustain larger compressive loads. In low-density structures, the slender struts are more susceptible to local buckling and plastic deformation, resulting in lower yield and plateau loads. In high-density structures, the thicker struts and more stable nodal connections preserve greater load-bearing capacity and better structural integrity during compression. Overall, increasing relative density effectively enhances the axial stiffness, yield strength, and plateau load-bearing capacity of uniform Octet LCSs, although it also subjects the structures to higher stress levels during compression.

#### 5.1.2. Effect of Gradient Distribution

**Figure 8:**
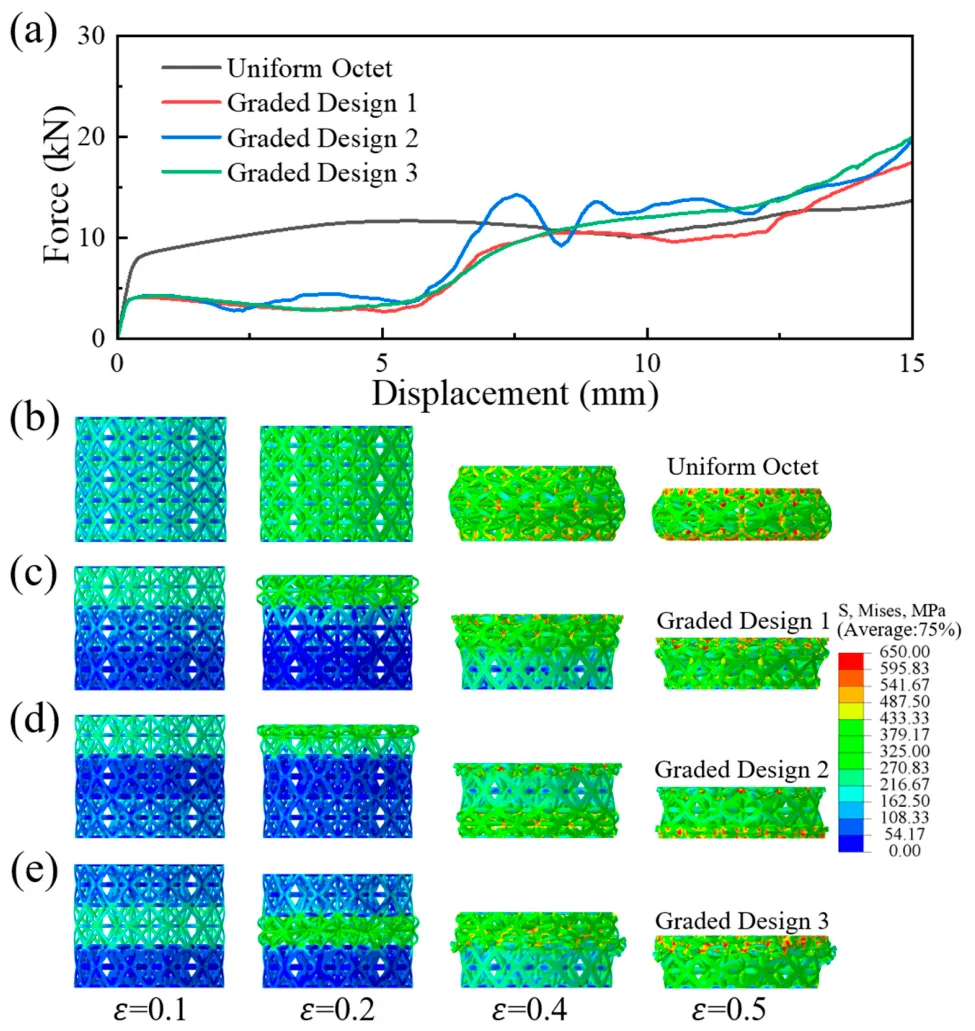
(**a**) Force–displacement curves and (**b**–**e**) deformation processes and stress distributions of the graded Octet LCSs under vertical compression.

Figure 8 presents the force–displacement curves and deformation processes and stress distributions of the uniform and graded Octet LCSs with the same average relative density under vertical compression. The gradient design clearly changes the compressive response and deformation mode. Compared with the uniform structure, the graded structures have a lower load-bearing capacity at the beginning of compression because the low-density layer has smaller strut diameters and lower local stiffness and therefore yields and collapses first. As the displacement increases, the medium- and high-density layers successively engage in load bearing, producing distinct stagewise rises and fluctuations in the force–displacement curves. By contrast, the uniform Octet LCS has the same density in all layers, so the layers carry load more synchronously; after initial yielding, the curve shows a relatively stable plateau and the deformation is more uniform. Thus, the gradient design does not simply increase the initial load-bearing capacity. Instead, it controls the yielding sequence of the density layers and produces layer-by-layer collapse and staged energy absorption during compression [53,55].

Comparison of the three graded structures shows that the sequence of the density layers further affects load-bearing stability and stress distribution. Changing the position of the low-density layer shifts the location of initial yielding and local collapse, thereby altering the plateau response and the magnitude of load fluctuations. Some graded structures exhibit pronounced load peaks and oscillations in the middle and later stages, indicating that local stiffness and stress concentration increase when the higher-density layers begin to carry load. The stress contours show that, at the beginning of compression, high stresses are concentrated mainly in the low-density layer that deforms first and in the adjacent nodal regions. As strain increases, the high-stress regions progressively extend to the other layers until the entire structure enters densification. Overall, graded Octet LCSs achieve a more controllable deformation path through sequential yielding of the density layers, allowing continuous load bearing and energy absorption over a wide displacement range. Within the three-layer configurations examined, these results demonstrate that rearranging the fixed density layers can regulate the location of initial yielding, the progressive collapse sequence, and the vertical compression response.

### 5.2. Transverse Loading Mechanical Properties

#### 5.2.1. Effect of Relative Density

**Figure 9:**
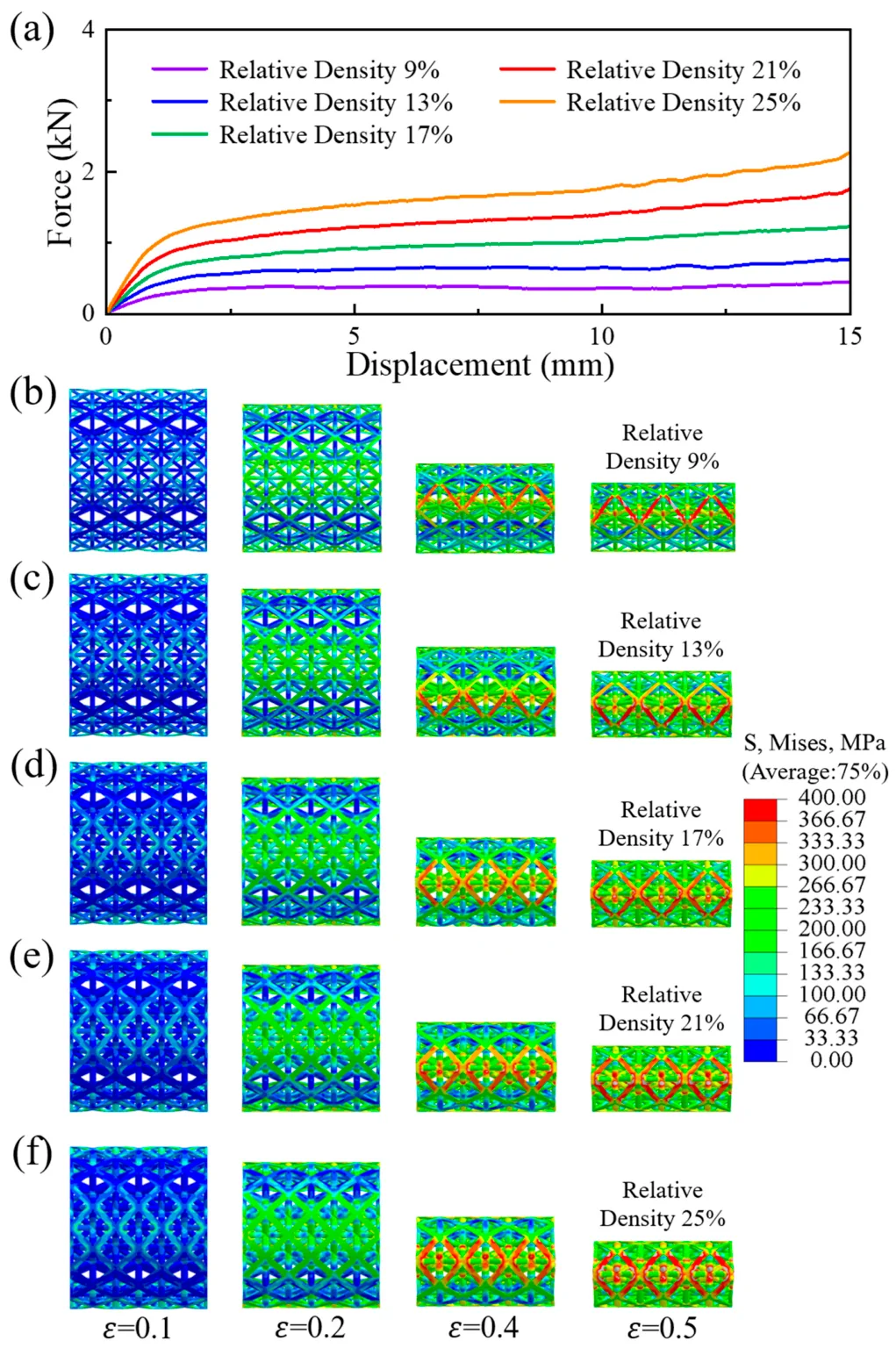
(**a**) Force–displacement curves and (**b**–**f**) deformation processes and stress distributions of the uniform Octet LCSs with different relative densities under transverse compression.

Figure 9 presents force–displacement curves and deformation processes and stress distributions of the uniform Octet LCSs with different relative densities under transverse compression. Compared with vertical compression, the load-bearing capacity is substantially lower under transverse compression because the LCS undergoes more pronounced global bending and local ovalization, and the lattice struts resist the external load mainly through bending, rotation, and local deformation. As the relative density increases from 9% to 25%, the transverse load-bearing capacity increases and the force–displacement curves shift upward, demonstrating that relative density also strongly affects the transverse mechanical response. A higher relative density corresponds to larger strut diameters, which increase the bending stiffness of the struts and the strength of the nodal connections, enabling the structure to carry higher loads at the same displacement. The curves for all densities rise relatively continuously and do not exhibit the extended plateau observed in vertical compression, indicating that transverse compression is governed mainly by global geometric deformation and local strut bending.

The stress contours in Figure 9b–f show similar deformation processes for all relative densities under transverse compression. At the beginning of compression, stress concentrations first develop near the contact regions between the specimen and the platens, after which the high-stress regions spread toward the middle and into adjacent struts. As the compressive strain increases, the cylindrical cross-section is progressively flattened, the lattice pores shrink, and some struts undergo pronounced bending and plastic deformation, followed by increasingly strong local contact at later stages. Owing to their slender struts, the low-density structures have lower transverse stiffness, stress levels, and load-bearing capacity. In the high-density structures, thicker struts maintain better structural integrity during compression, and the more extensive high-stress regions indicate that they carry larger transverse loads. Overall, increasing relative density effectively enhances the transverse stiffness and load-bearing capacity of uniform Octet lattice cylindrical shells. Nevertheless, the transverse response remains dominated by bending and local flattening, and the load-bearing level is substantially lower than that under vertical compression.

#### 5.2.2. Effect of Gradient Distribution

**Figure 10:**
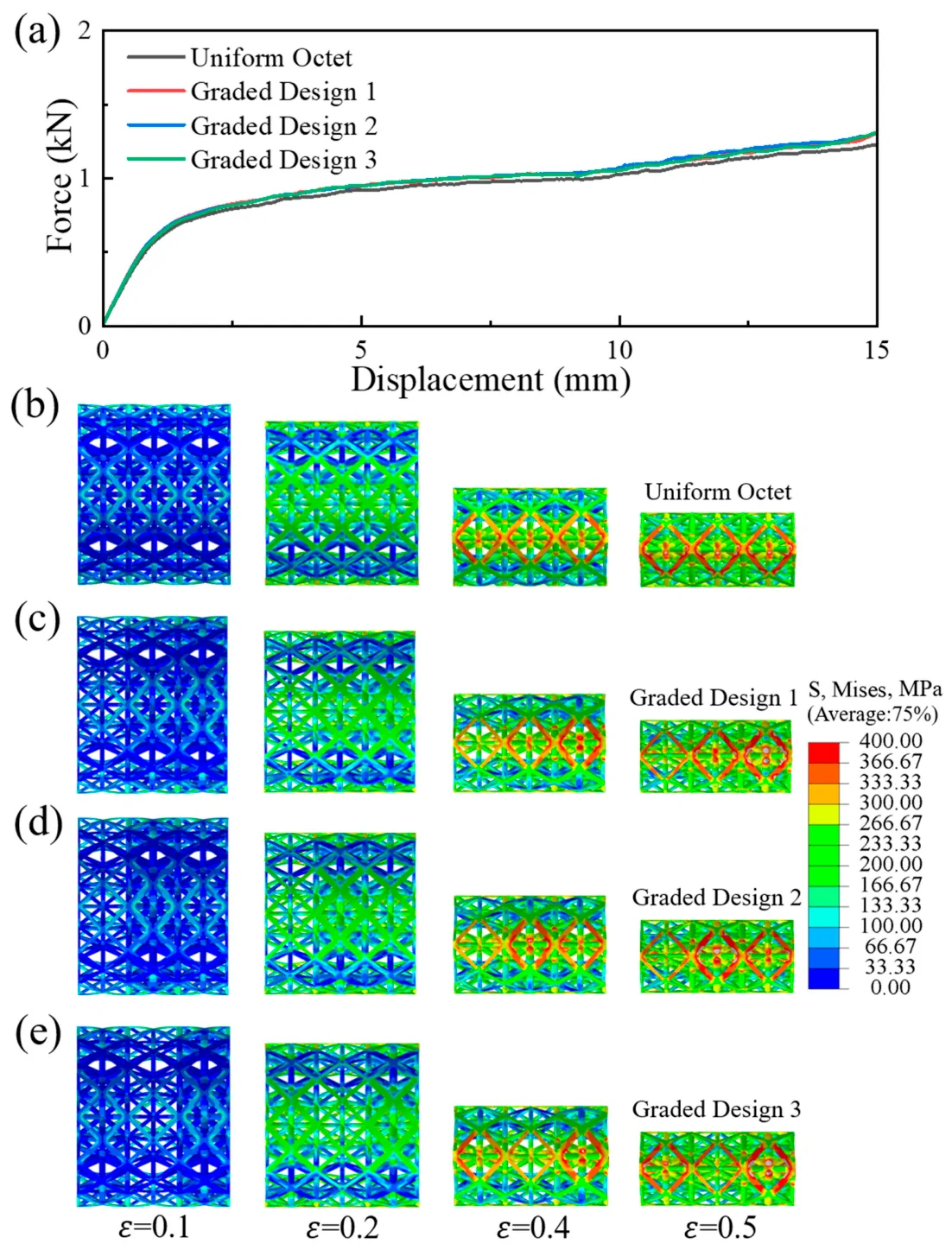
(**a**) Force–displacement curves and (**b**–**e**) deformation processes and stress distributions of the graded Octet LCSs under transverse compression.

Figure 10 presents the force–displacement curves and deformation processes and stress distributions of the uniform and graded Octet LCSs with the same average relative density under transverse compression. Unlike the vertical compression results, the gradient design has a relatively weak effect on transverse load-bearing capacity. The force–displacement curves of the four structures nearly overlap and all rise rapidly at first and then increase slowly. This indicates that, under transverse compression, the global response is governed mainly by flattening of the cylindrical cross-section, strut bending, and local contact deformation, while the vertical sequence of the density layers has only a limited influence on the overall transverse load-bearing capacity. Because all graded structures have the same average relative density and therefore nearly the same total material volume fraction, they exhibit similar overall stiffness and load-bearing levels during transverse compression.

The stress contours and deformation processes in Figure 10b–e show pronounced cross-sectional flattening in both the uniform and graded structures during transverse compression. As the compressive strain increases, the lattice pores progressively close, strut bending and mutual contact intensify, and the high-stress regions concentrate mainly in the compressed contact zones and in struts undergoing pronounced bending. Although the global force–displacement responses of the graded structures differ only slightly, their local stress distributions are still affected by the density sequence. Because of their smaller strut diameters, the low-density layers are more prone to local bending and deformation, whereas the high-density layers provide stronger support, maintain greater local stiffness, and carry higher stresses. Thus, gradient design primarily changes the local deformation path and the locations of stress concentrations during transverse compression rather than substantially altering the overall load-bearing capacity. In general, the mechanical response of Octet LCSs under transverse loading is dominated by global geometric flattening. The effect of layer sequence on load-bearing performance is weaker than under vertical compression, although it can still be used to tailor local deformation and stress distribution.

### 5.3. Key Performance Parameters

**Figure 11:**
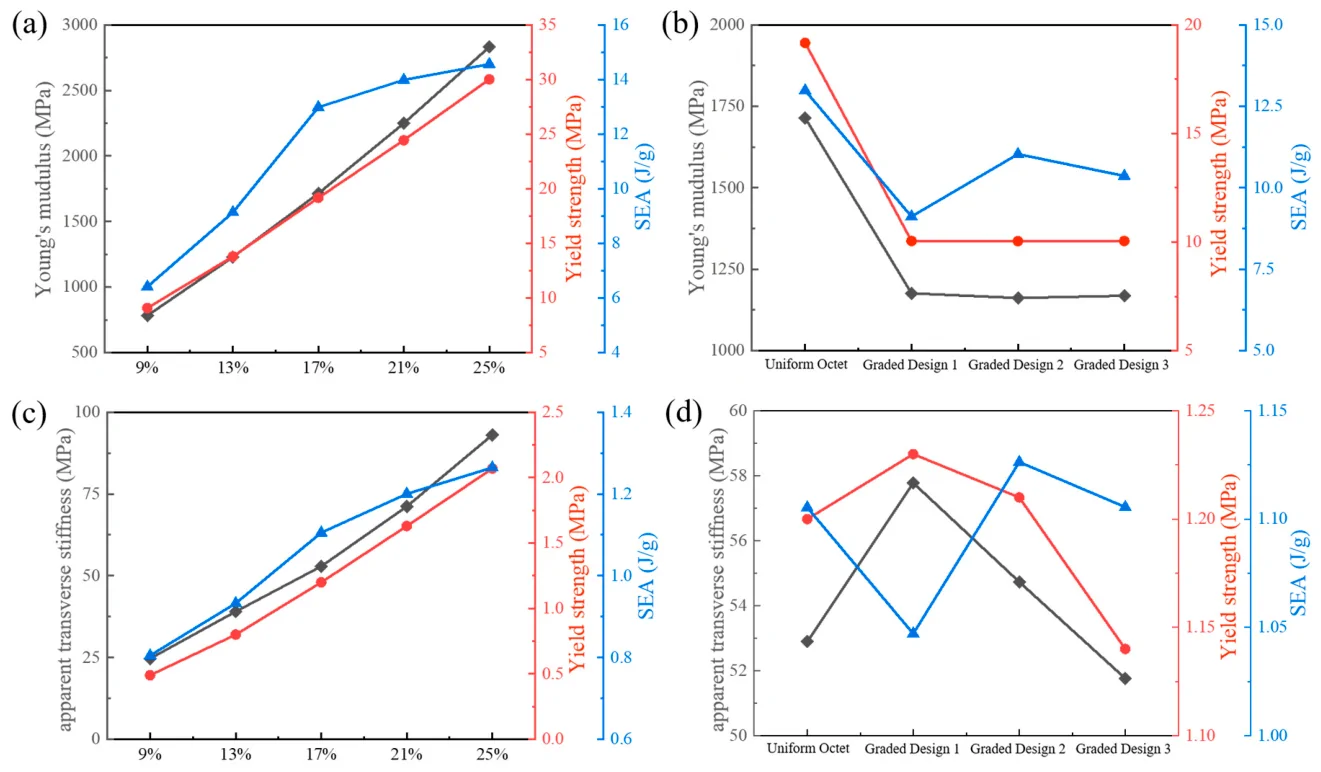
Comparison of Young’s modulus under vertical compression, apparent transverse stiffness under transverse compression, yield strength, and SEA of Octet LCSs under quasi-static compression: (**a**) uniform Octet LCSs with different relative densities under vertical loading; (**b**) uniform and graded Octet LCSs with the same relative density under vertical loading; (**c**) uniform Octet LCSs with different relative densities under transverse loading; (**d**) uniform and graded Octet LCSs with the same relative density under transverse loading.

Young’s modulus, yield strength, and SEA were determined from the compressive responses. SEA was calculated from the area under the force–displacement curve up to a displacement of 15 mm. The design mass was used for SEA normalization in all cases, including both experiment–simulation comparisons and numerical analyses. For the uniform and graded structures, yield strength was defined as the nominal compressive stress at the first departure from the initial linear-elastic response. Figure 11 compares the principal mechanical parameters of the Octet LCSs under different loading directions, including Young’s modulus, yield strength, and SEA.

For the uniform structures under vertical compression in Figure 11a, at relative densities of 0.09, 0.13, 0.17, 0.21, and 0.25, Young’s modulus was 782.8 MPa, 1228.8 MPa, 1714.5 MPa, 2250.0 MPa, and 2832.7 MPa respectively; yield strength was 9.1 MPa, 13.8 MPa, 19.2 MPa, 24.5 MPa, and 30.0 MPa respectively; and SEA was 6.41 J/g, 9.14 J/g, 12.99 J/g, 13.99 J/g, and 14.56 J/g respectively. Under transverse compression in Figure 11c, the corresponding apparent transverse stiffness values were 24.7 MPa, 39.1 MPa, 52.9 MPa, 71.2 MPa and 93.1 MPa, the yield strengths were 0.49 MPa, 0.80 MPa, 1.20 MPa, 1.63 MPa and 2.07 MPa, and the SEA values were 0.81 J/g, 0.93 J/g, 1.11 J/g, 1.20 J/g and 1.27 J/g. Although the density-dependent trends were similar, the mechanical parameters under vertical compression remained substantially higher than those under transverse compression over the entire density range. This pronounced directional difference is attributed to the distinct load-transfer mechanisms: vertical compression is dominated by axial stretching and compression of the inclined struts, whereas transverse loading is governed mainly by cross-sectional flattening, strut bending, and local contact.

Figure 11b compares the uniform and graded structures under vertical compression at the same average relative density of 0.17. The uniform structure exhibited a Young’s modulus of 1714.5 MPa, a yield strength of 19.2 MPa, and an SEA of 12.99 J/g. The corresponding average values for the graded structures were 1168.8 MPa, 10.1 MPa, and 10.17 J/g, respectively. Compared with the uniform structure, Young’s modulus and SEA decreased by 31.8% and 21.7%, respectively. The first-yield value of the graded structures was numerically lower, because it represents initial yielding of the low-density layer. Among the graded configurations, Young’s modulus and yield strength varied by only 1.2% and 0.1% respectively. By contrast, the SEA ranged from 9.12 J/g for Graded design 1 to 11.03 J/g for Graded design 2, with the latter being 21.0% higher than that of Graded design 1. Therefore, the layer sequence had little effect on the initial stiffness and yield strength of the graded structures but had a measurable influence on their vertical energy-absorption response.

Under transverse compression at the same average relative density of 0.17 in Figure 11d, the uniform structure had an apparent transverse stiffness of 52.9 MPa, a yield strength of 1.2 MPa, and an SEA of 1.11 J/g. For the graded structures, apparent transverse stiffness ranged from 51.8 MPa to 57.8 MPa, yield strength from 1.14 MPa to 1.23 MPa, and SEA from 1.05 J/g to 1.13 J/g. Relative to the uniform structure, the largest absolute change was 9.2%, observed for the apparent transverse stiffness of Graded design 1, and the changes in all three mechanical parameters remained below 10%. These limited variations confirm that the gradient-layer sequence has only a minor effect on global transverse load-bearing and energy-absorption performance, although it still modifies local stress and deformation distribution. Overall, relative density primarily determines the magnitude of the macroscopic properties, whereas gradient design mainly regulates the yielding sequence, collapse path, plateau fluctuations, and local stress distribution.

## 6. Conclusions

A uniform Octet LCS and three graded Octet LCSs were designed and fabricated in this study. Vertical and transverse quasi-static compression experiments combined with finite element simulations were conducted to systematically investigate the effects of relative density and gradient-layer sequence on compressive performance, deformation modes, and energy-absorption behavior. The main conclusions are as follows:

(1) For uniform Octet LCSs, relative density is the primary factor governing the macroscopic mechanical properties. As the relative density increased from 9% to 25%, Young’s modulus, yield strength, and SEA increased markedly under both vertical and transverse compression, demonstrating that larger strut diameters and a greater material volume fraction effectively enhance structural stiffness, load-bearing capacity, and energy-absorption performance.
(2) Under vertical compression at the same average relative density, the uniform LCS exhibited higher stiffness and SEA, whereas the graded LCSs exhibited a more controllable sequential layer-by-layer collapse by sacrificing part of their load-bearing capacity. Among the graded designs, Young’s modulus and yield strength differed by only 1.2% and 0.1%, while Graded design 2 achieved 21.0% higher SEA than Graded design 1, indicating that the layer sequence mainly affects energy absorption.
(3) Under transverse compression at the same average relative density, the response was governed mainly by flattening of the cylindrical cross-section, strut bending, and local contact. The differences in the principal mechanical parameters between the uniform and graded structures did not exceed 10%, indicating that the gradient-layer sequence had only a limited effect on the overall transverse response, although it could still regulate local stress and deformation.

In summary, relative density determines the principal load-bearing level of Octet LCSs, whereas, within the three-layer configurations examined, rearranging the fixed density layers regulates sequential yielding, local collapse, and the energy-absorption process. These findings provide guidance for the layer-sequence design of lightweight protective structures, cushioning and energy-absorbing devices, and curved lattice shells.

## Figures and Tables

**Table 1:** Geometric dimensions of the as-designed Octet LCSs.

Sample		Uniform Octet					Graded Octet		
							Graded-1	Graded-2	Graded-3
$\bar{\rho }$		0.09	0.13	0.17	0.21	0.25	0.17		
$L_{1}$ (mm)		20					20		
$L_{2}$ (mm)		30					30		
H (mm)		25					25		
Strut diameter D (mm)	Layer 1	0.440	0.538	0.624	0.704	0.778	0.440	0.440	0.624
	Layer 2						0.624	0.778	0.440
	Layer 3						0.778	0.624	0.778

## Data Availability

The original contributions presented in this study are included in the article. Further inquiries can be directed to the corresponding authors.
